# Role of Diagnostic Hysteroscopy in Abnormal Uterine Bleeding and its Histopathologic Correlation

**DOI:** 10.4103/0974-1216.71617

**Published:** 2009

**Authors:** Sheetal G Patil, S B Bhute, S A Inamdar, Neema S Acharya, Deepti S Shrivastava

**Affiliations:** Senior Resident, Department of Obstetrics and Gynecology, Datta Meghe Institute of Medical Sciences, Wardha - 442 004, Maharashtra, India; 1,2 Professor, Department of Obstetrics and Gynecology, Datta Meghe Institute of Medical Sciences, Wardha - 442 004, Maharashtra, India; 3,4 Associate Professor, Department of Obstetrics and Gynecology, Datta Meghe Institute of Medical Sciences, Wardha - 442 004, Maharashtra, India

**Keywords:** Abnormal uterine bleeding, dilatation and curettage, hysteroscopy

## Abstract

**Aims and Objectives:**

To study the accuracy of hysteroscopy in evaluation of abnormal uterine bleeding and to correlate hysteroscopic findings with histopathologic findings.

**Materials and Methods:**

A prospective study was carried out in the Department of Obstetrics and Gynecology at AVBRH and JNMC, Sawangi (M), Wardha, from May 2006 to September 2008. One hundred cases were selected for this study from patients of age group 18 and above, who were admitted with the history of abnormal uterine bleeding. Hysteroscopic examination was done in all patients post-menstrually, whenever possible, except in those cases where menstrual cycles were grossly irregular or patients came with continuous bleeding per vaginum. The patients then underwent dilatation and curettage and endometrium was sent for histopathologic examination. The correlation between findings on hysteroscopy and histopathologic examination was tabulated.

**Results:**

Following were the findings on hysteroscopy: proliferative 34%, secretary 16%, hyperplasia 18%, atrophic 8%, endometrial polyp 9%, submucous myoma 11%, carcinoma of endometrium 03%, misplaced Cu-T 1%.

**Conclusion:**

In patients with abnormal uterine bleeding, hyster-oscopy provides more accurate dia-gnosis than dilatation and curettage.

## INTRODUCTION

Menstrual dysfunction is the cause of discomfort, inconvenience and disruption of healthy lifestyle, which affects millions of women in both developed and developing world.[1] Until recent times, usual method of evaluating this symptom was dilatation and curettage. But this detects the cause in less than 50% of the cases.

Hysteroscopy offers a valuable extension of the gynecologist’s armamentarium. It can improve the diagnostic accuracy and can permit better treatment of uterine diseases. After hysteroscopy, the elective surgery of the patient can be planned better.[2] Use of hysteroscopy in abnormal uterine bleeding is almost replacing blind curettage, as it “sees” and “decides” the cause. This is because the uterine cavity can be observed and the area in question can be curetted. In fact, it is an eye in the uterus.[3]

The aims and objectives of this study were to study the accuracy of hysteroscopy in evaluation of abnormal uterine bleeding and to correlate hysteroscopic findings with histopathologic findings.

### Aims and objectives

1. To study the accuracy of hysteroscopy in evaluation of abnormal uterine bleeding.
2. To correlate hysteroscopic findings with histopathologic findings.

## MATERIALS AND METHODS

The study was carried out in the Department of Obstetrics and Gynecology at AVBRH and JNMC, Sawangi (M), Wardha, from May 2006 to September 2008. One hundred cases were selected for this study from the patients of age group 18 and above, who were admitted with the history of abnormal uterine bleeding; excluding any demonstrable pelvic pathology like fibroids, cancer of cervix or vagina or endometrium on clinical examination and having active pelvic infection, coagulation disorder, thyroid disease, pregnancy, patients on hormonal drugs like tamoxifen, patients with active profuse uterine bleeding and history of recent intrauterine perforation.

Patients were subjected to a detailed history and examination, and investigations were done. Hysteroscopic examination was done in all patients post-menstrually, whenever possible, except in those cases where menstrual cycles were grossly irregular or patients came with continuous bleeding per vaginum (PV).

The patients then underwent dilatation and curettage and endometrium was sent for histopathologic examination. The correlation between findings on hysteroscopy and histopathologic examination was tabulated.

Further management of the patient was decided according to age, parity, severity of the disease, hysteroscopic and histopathologic report. Data were recorded on a predesigned proforma.

## RESULTS

In the study, the age of patients varied from 20 to 65 years. Abnormal uterine bleeding was most prevalent among women of two age groups, 26–30 years and 41–45 years (22%).

The commonest affected patients were para 3 or more (33%) and least affected were para 1 (10%). Exactly 59% of the cases belonged to Middle socioeconomic strata, 18% to high socioeconomic strata and 23% of the cases belonged to poor socioeconomic strata. Also, 68% of the population was from rural set-up mostly because 70% of Indian population lives in rural area and ours is a rural hospital, draining mainly rural population.

Fourteen percent of the patients were hypertensive, 6% had history of tuberculosis, 5% were diabetic and 5% of the patients were having sickle cell disease (Vidarbha being a sickle cell zone). Patients with coagulation disorder and thyroid disorder were excluded from this study.

Table 1 represents the incidence of various bleeding patterns in the present study.

**Table 1:** Abnormal uterine bleeding pattern

Abnormal uterine bleeding pattern	No. of cases (100)	Percentage
Menorrhagia	25	25
Polymenorrhea	06	06
Polymenorrhagia	13	13
Metrorrhagia	05	05
Menometrorrhagia	01	01
Continuous bleeding PV	09	09
Metropathia hemorrhagica	02	02
Hypomenorrhea	15	15
Oligomenorrhea	17	17
Post menopausal bleeding	07	07

In the present study, 55% patients had abnormal uterine bleeding for more than 3 months to 1 year duration. Eighteen percent patients presented after 1 year duration of Abnormal Uterine Bleeding (AUB), 14% presented after 1–3 months duration because uterine bleeding is mostly neglected in our country.

We observed that infertility (35%) was the most commonly associated complaint, followed by pain in abdomen (26%) and dysmenorrhea (18%).

Out of 100 patients, 10% patients had undergone D and C in the past, 9% had undergone cesarean section and 2% had undergone suction and evacuation. Five percent of the patients had hemoglobin less than 6 g%, 28% patients had hemoglobin between 6 and 8g%. These patients required blood transfusion prior to hysteroscopy and curettage.

In 62% of the cases, cervix was found to be normal, in 21% cases hypertrophied and in 17% cases eroded. On per vaginum examination, the most common finding was normal size uterus (50%), followed by 6 week size uterus (30%). Uterine size of more than 6 weeks or any detectable pathology like fibroid uterus, cervical polyp and carcinoma were excluded from this study.

Out of 100 patients, 74% had normal pap smear report and 26% had inflammatory pap smear report. Sixty-two percent of the patients were having normal ultrasonographic findings. Endometrial hyperplasia (15%) was the most commonly detected pathology, followed by fibroid uterus (13%), on ultrasonography. Adnexal mass was found in seven cases, endometrial polyp in two cases while endometrial carcinoma was suspected in one case. Hysteroscopy was carried out in 100 cases in the operation theater. Intravenous pethidine 50 mg + inj. phenargan 25 mg intramuscularly were used in 21% cases and general anesthesia in 79% cases because concomitant laparoscopy was done along with hysteroscopy or due to the anxiety in some patients, General anaesthesia (GA) was used.

In the present study, the distending medium used was normal saline.

All the patients underwent diagnostic, hysteroscopic-guided curettage and the tissue was sent for histopathologic diagnosis.

The hysteroscopic findings were correlated with histopathologic findings.

Out of 42 cases of proliferative endometrium on histopathology, in 41 cases, proliferative endometrium was deduced on hysteroscopy. Out of 41 cases, 33 were confirmed on hysteroscopy as proliferative endometrium (34 cases of proliferative endometrium diagnosed by hysteroscopy are shown in Table 4. Other seven cases are not shown in the table. As with other findings, three cases of endometrial polyp, three cases of myoma and one case of misplaced Cu-T were missed by histopathology but were diagnosed on hysteroscopy along with proliferative endometrium, which are not included in this table due to technical problems). On hysteroscopy, the remaining five cases (14.7%) showed secretory endometrium and three cases (8.8%) showed simple hyperplasia without atypia.

Out of 22 cases of secretory endometrium on histopathology, 17 were diagnosed on hysteroscopy (one case which was associated with submucous myoma finding on hysteroscopy is not shown in the table). Out of these 17 cases, 12 were confirmed as secretory endometrium on histopathology. On hysteroscopy, the remaining three cases (18.75%) showed proliferative endometrium and one case (6.25%) each showed simple hyperplasia without atypia and tuberculous endometritis.

Twenty cases of hyperplasia were diagnosed on histopathology, but 21 cases were suspected of hyperplasia on hysteroscopy (three cases are not included in the table as these cases of hyperplasia were associated with one case of endometrial polyp and two cases of submucous myoma). Out of 15 cases of hyperplasia on hysteroscopy, 9 showed simple hyperplasia without atypia, 3 showed simple hyperplasia with atypia, 3 showed complex hyperplasia without atypia and 1 case showed complex hyperplasia with atypia, 3 cases showed proliferative endometrium, 2 cases showed secretory endometrium and 1 case showed tuberculous endometritis.

Out of 20 cases of hyperplasia on histopathology, 13 cases were of simple hyperplasia without atypia, 3 cases were of simple hyperplasia with atypia, 3 cases were of complex hyperplasia without atypia and 1 case was of complex hyperplasia with atypia.

Out of three cases suspected as carcinoma endometrium, which was diagnosed as hyperplasia with areas of ulceration, increased vascularity, hemorrhage or any growth, two cases (66%) were confirmed on histopathology. One showed adenocarcinoma of uterus and the other one showed papillary serous carcinoma of endometrium. One case of suspected carcinoma was diagnosed as proliferative endometrium.

On hysteroscopy, eight cases showed atrophic endometrium, out of which three cases (37.5%) were confirmed on histopathology; in two cases, no endometrial tissue was obtained. One case showed proliferative endometrium, one case showed secretory endometrium and in one case tuberculous endometritis was diagnosed.

Hysteroscopy showed 11 cases of submucous myoma; only 1 case was confirmed on histopathology. But all the cases of fibroid were confirmed by post-hysteroscopy. Six cases were confirmed on hysterectomy specimen; in two cases myomectomy was done, in two cases uterine artery embolization was done and one case was confirmed by laparoscopy.

Hysteroscopy showed nine cases of endometrial polyp, out of which five were confirmed on histopathology. Other four cases were confirmed after polyp removal done by operative hysteroscopy (done outside).

After confirming the diagnosis, treatment was given to the patients. Hysterectomy was done in 21 patients. In two patients, myomectomy was done. Seven submucosal fibroids and three endometrial polyps were removed by operative hysteroscopy (done outside). In one case, misplaced Cu-T was removed. In two cases synechie in uterine walls was broken and Cu-T was put to prevent further adhesions. In two patients, uterine artery embolization was done in intramural fibroid. In six patients, thermal balloon ablation was done. So, radical treatment was avoided in 23 patients. Thirty-three patients were put on hormonal treatment according to endometrial histopathology report. Thus, hysteroscopy played an important role in the treatment of abnormal uterine bleeding. Two patients had uterine perforation in the complications of hysteroscopy. But both were small rents and did not require any treatment expect monitoring. The time required for the whole procedure was 20–30 min.

## DISCUSSION

Abnormal uterine bleeding is one the most frequently encountered conditions in gynecology. As quoted by Devi and Menon,[4] the incidence is 30–40% of all gynecological cases.

In this prospective study, 100 women between 20 and 65 years of age who presented with complaints of abnormal uterine bleeding pattern had undergone two modalities of investigations to reach a conclusion - diagnostic hysteroscopy and endometrial histpathology report.

This study was undertaken to correlate the hysteroscopic findings with histopathologic report. The results of various authors is shown in Tables 1 to 10.

**Table 2:** Hysteroscopic findings

Findings	No. of cases (100)	Percentage
Proliferative	34	34
Secretory	16	16
Hyperplasia	18	18
Atrophic	08	08
Endometrial polyp	09	09
Submucous myoma	11	11
Carcinoma endometrium	03	03
Misplaced Cu-T	01	01

**Table 3:** Histopathologic report

Histopathologic report	No. of cases (100)	Percentage
Proliferative	42	42
Secretory	22	22
Simple hyperplasia without atypia	13	13
Simple hyperplasia with atypia	03	03
Complex hyperplasia without atypia	03	03
Complex hyperplasia with atypia	01	01
Endometrial polyp	05	05
Fibroid polyp	01	01
Carcinoma endometrium	02	02
Tuberculous endometritis	03	03
No endometrial tissue obtained	02	02
Atrophic endometrium	03	03

**Table 4:** Correlation between hysteroscopic finding and histopathologic report

Hysteroscopic finding	Histopathologic report												
	Proliferative	Secretory	SH without A	SH with A	CH without A	CH with A	Atrophic	TB endometritis	End. polyp	Fibroid polyp	No endo. tissue	Ca endo.	Total
Proliferative	26	05	03	00	00	00	00	00	00	00	00	00	34
Secretory	03	11	01	00	00	00	00	01	00	00	00	00	16
Hyperplasia	03	02	07	01	03	01	00	01	00	00	00	00	18
Atrophic	01	01	00	00	00	00	03	01	00	00	02	00	08
Ca. endometrium	01	00	00	00	00	00	00	00	00	00	00	02	03
Submucous myoma	04	03	02	01	00	00	00	00	00	01	00	00	11
Endo. polyp	03	00	00	01	00	00	00	00	05	00	00	00	09
Misplaced Cu-T	01	00	00	00	00	00	00	00	00	00	00	00	01
Total	42	22	13	03	03	01	03	03	05	01	02	02	100

SH - simple hyperplasia; CH - complex hyperplasia; A - atypia; TB - tuberculous; endo. - endometrial, Ca - carcinoma

**Table 5:** Investigators reporting normal uterine cavity in their studies

Author	Year	Patients with normal uterine cavity (%)
Gribb[6]	1960	57
Silander[7]	1963	53.33
Seigler[8]	1976	50
Sheth[9]	1989	44
de Jong[10]	1990	47.36
Anuradha Panda[5]	1999	46.6
Jyotsana[11]	2004	34
Present study	2008	50

**Table 6:** Reports of hyperplasia by different studies

Author	Year	Incidence of hyperplasia (%)
Silander[8]	1963	6.66
Dexus[15]	1981	21
Sheth[9]	1989	26
Anuradha Panda[5]	1999	28.3
Jyotsana[11]	2004	22.66
Present study	2008	20

**Table 7:** Incidence of myoma in different studies

Author	Year	Incidence of myoma (%)
Valle[14]	1981	14.33
Mencalgia[17]	1987	9.6
de Jong[10]	1990	11.18
Anuradha Panda[5]	1999	6.6
Jyotsana[11]	2004	17.33
Present study	2008	11

**Table 8:** Incidence of endometrial polyp reported by different authors

Author	Year	Incidence of endometrial polyp (%)
Silander[7]	1963	6.66
Mencalgia[17]	1987	9.8
de Jong[10]	1990	7.8
Anuradha Panda[5]	1999	10
Acharya Veena[16]	2003	12
Jyotsana[11]	2004	20
Present study	2008	9

**Table 9:** Incidence of atrophic endometrium in different studies

Author	Year	Incidence of atrophic endometrium (%)
Sciarra[19]	1977	5
Alawani[20]	1983	9.67
Mencalgia[17]	1987	12
Anuradha Panda[5]	1999	5
Present study	2008	8

**Table 10:** Incidence of carcinoma endometrium in different studies

Author	Year	Incidence of carcinoma endometrium (%)
Sciarra[19]	1977	1.26
Valle[14]	1981	0.53
de Jong[10]	1990	3.3
Jyotsana[11]	2004	1.3
Present study	2008	2

### Group I: Patients with proliferative endometrium

In 41 patients, endometrium was pink, smooth and thin, appearing to be of proliferative type. The same was confirmed by histopathology in 33 patients. Histology of the endometrial curetting revealed proliferative endometrium with tall columnar cells and pseudostratification. Findings were different in eight cases. Diagnostic accuracy of hysteroscopy for proliferative endometrium was 81%. Sensitivity, specificity, positive predictive value and negative predictive value of hysteroscopy for proliferative endometrium compared to histopathology were 78.57, 86.2, 80.48 and 84.74%, respectively.

### Group II: Patients with secretory endometrium

Hysteroscopy proved uterine cavity to be normal with orange, undulating and thick endometrium appearing to be secretory endometrium in 17 cases. It was confirmed in 12 cases. Diagnostic accuracy of hysteroscopy for secretory endometrium was 71%. So, sensitivity, specificity, positive predictive value and negative predictive value of hysteroscopy for secretory endometrium were 54.54, 93.58, 70.58 and 87.95%, respectively.

Overall diagnostic accuracy for diagnosing normal endometrium was 85.93% for hysteroscopy. Panda *et al*,[5] had reported diagnostic accuracy for normal endometrium as 92.5%.

### Group III: Patients with hyperplastic endometrium

In these patients, the endometrium appeared to be thickened, edematous and undulating. There were 21 patients with this hysteroscopic finding. This finding was consistent with histology of the endometrium in 15 cases and differed in 6 cases.

So, hysteroscopic diagnostic accuracy for hyperplasia was 72%. So, sensitivity, specificity, positive predictive value and negative predictive value of hysteroscopy for hyperplasia were 75, 92.5, 71.4 and 93.67%, respectively.

Loverro *et al*,[12] stated the sensitivity, specificity, positive predictive value and negative predictive value as 98, 95, 63 and 99%, respectively, for endometrial hyperplasia.

Arslan *et al*,[13] did hysteroscopy in 216 premenopausal and 114 postmenopausal women for diagnosing hyperplasia. The positive predictive value was 71.4% and negative predictive value was 95.4% in diagnosis.

Diagnostic accuracy of hysteroscopy for endometrial hyperplasia was 68.2, 71.4 and 76.4% in a series reported by Valle *et al*,[14] Seth *et al*,[9] and Panda *et al*,[5] respectively.

### Group IV: Submucous myoma

A white-colored bulge, round in shape, with a smooth surface, which was diagnosed on hysteroscopy as submucous leiomyoma, was found in 11 patients. Only one case was confirmed on histopathology.

Diagnostic accuracy of hysteroscopy for submucous fibroid was only 9.09%. But considering the final diagnosis, diagnostic accuracy of hysteroscopy was 100%. So, sensitivity, specificity, positive predictive value and negative predictive value of hysteroscopy for fibroid compared with histopathology were 100, 89.89, 9.09 and 100%, respectively. But compared with final diagnosis, sensitivity, specificity, positive predictive value and negative predictive value of hysteroscopy for fibroid were 100% each. Similar findings were reported by Panda[5] and Veena.[16] But Valle[14] and Sheth[9] had reported 88 and 81%, respectively, of diagnostic accuracy.

### Group V: Patients with endometrial polyp

In 9 (9%) patients on hysteroscopy, small growths in the uterine cavity, which were soft, oval, pedunculated with a smooth surface were seen. These growths appeared as endometrial polyps. Histopathology report confirmed the findings in five cases.

Diagnostic accuracy of hysteroscopy for endometrial polyp was 62% when compared to histopathology. But considering the final diagnosis, diagnostic accuracy was 100%. So, sensitivity, specificity, positive predictive value and negative predictive value of hysteroscopy for endometrial polyp compared to histopathology were 100, 95.78, 55.55 and 100%, respectively. But compared to final diagnosis, sensitivity, specificity, positive predictive value and negative predictive value of hysteroscopy for endometrial polyp were 100% each. Haller *et al*,[18] had reported sensitivity and specificity of 100 and 96.7%, respectively. Anuradha Panda[5] had reported diagnostic accuracy of 100% in diagnosing polyp. Acharya Veena[16] had obtained sensitivity, specificity, positive predictive value and negative predictive value of hysteroscopy for endometrial polyp as 100% each. But Valle[14] and Seth[9] had obtained a diagnostic accuracy of 88.6 and 81.8%, respectively.

### Group VI: Atrophic endometrium

In 8 (8%) patients, the endometrium appeared flat, thin and fragile. At some points; petechie and hemorrhages were present. The tubal ostia were very prominent. The picture was suggestive of atrophic endometrium, which was also confirmed by histopathology in three cases and endometrium was not obtained in two cases.

Diagnostic accuracy of hysteroscopy was 63%. So, sensitivity, specificity, positive predictive value and negative predictive value of hysteroscopy for atrophic endometrium were 100, 96.84, 62.5 and 100%, respectively. This correlated with the report of Panda *et al*.[5] Haller *et al*,[18] had reported sensitivity and specificity of 100 and 97%, respectively.

### Group VII: Carcinoma endometrium

In 3 (3%) patients, hyperplasia, with polypoidal growth, with areas of ulceration, hemorrhage and increased vascularity were labeled as carcinoma endometrium on hysteroscopy. Two cases were confirmed on histopathology.

Diagnostic accuracy of hysteroscopy was 67%. So, sensitivity, specificity, positive predictive value and negative predictive value of hysteroscopy for carcinoma endometrium were 100, 98.97, 66.66 and 100%, respectively.

Mencaglia[17] combined hysteroscopy with endometrial biopsy for diagnosing endometrial carcinoma and found nearly 100% accuracy in the diagnosis of endometrial neoplasia and its precursors.

But Haller *et al*,[18] had got a reduced sensitivity of 50% but better specificity of 100%. Valle[14] and Panda[5] had obtained diagnostic accuracy of 100% each. Hysteroscopy revealed a sensitivity, specificity, positive predictive value and negative predictive value of 100, 49.6, 81 and 100%, respectively, according to Pietro Litta *et al*.[21]

Tuberculous endometritis was the only condition missed by hysteroscopic viewing. Misplaced Cu-T was also detected by hysteroscopy with 100% accuracy.

So, to conclude, hysteroscopy was more accurate (100%) in identifying intrauterine pathologies like endometrial polyp, submucous myoma and misplaced Cu-T, than endometrial biopsy or dialatation and curettage alone.[4] Histopathology had 100% accuracy in diagnosing carcinoma endometrium. The diagnosis of hyperplasia, its types and carcinoma was only possible after histopathologic examination.

## CONCLUSION

Hysteroscopy is a valuable, simple, low-risk tech-nique which allows an adequate exploration of the uterine cavity under visual control.

It ensures speed and safety with the diagnosis and treatment. The results are immediately available.

In patients with abnormal uterine bleeding, hyster-oscopy provides the possibility of immediate diagnosis and prompt and effective treatment. It allows finding out the source of bleeding and perform a directed biopsy of the suspected area. It affords a more accurate diagnosis than dilatation and curettage for intrauterine pedunculated pathologies. But for hyperplasia and carcinoma endometrium, histopathology is 100% diagnostic.

Lesions like endometrial polyps and pedunculated fibromyomas can be removed under direct vision with the hysteroscope.

Diagnosis of endometrial atrophy is best made by hysteroscopy. Curettage does not always yield a posi-tive diagnosis of this condition and may even worsen the condition.

It is a very helpful technique in patients with intrauterine synechia. Since it can detect their presence, extension and nature, and these can also be removed under visual control with the hysteroscope only.

So it can be concluded that hysteroscopy offers an invaluable advantage of direct visualization of any abnormality within the uterine cavity. It does not substitute other diagnostic procedures; rather, it complements them. Hysteroscopy is a safe, simple, quick and economic technique, well-accepted by the patient, with great potential in gynecology.

Hysteroscopic-guided biopsy and histopathology are considered as the “new gold standard” in evaluating a case of abnormal uterine bleeding.
